# Intentional reimplantation of a mandibular premolar with severe apical root resorption: a case report demonstrating diagnostic complexity in adjacent teeth

**DOI:** 10.3389/froh.2025.1676465

**Published:** 2025-10-31

**Authors:** Marwa Ameen, Firas Elmsmari, Abdul Rahman Saleh

**Affiliations:** 1 Department of Clinical Sciences, College of Dentistry, Ajman University, Ajman, United Arab Emirates; 2 Center of Medical and Bio-allied Health Sciences Research, Ajman University, Ajman, United Arab Emirates

**Keywords:** root resorption, tooth replantation, endodontics, pulp vitality tests, cone-beam computed tomography, non-surgical root canal treatment, apical periodontitis, intentionalReplantation

## Abstract

**Purpose:**

This case report aimed to illustrate the clinical application of intentional reimplantation (IR) as a conservative and effective treatment strategy in cases of significant apical root resorption when surgical endodontics poses a risk to adjacent anatomical structures. Furthermore, it addressed the diagnostic challenge concerning the neighboring tooth, underscoring the importance of a comprehensive diagnostic evaluation to accurately differentiate between pathologies and prevent misdiagnosis.

**Case presentation:**

A 25-year-old woman presented with tooth #44 that had previously initiated root canal therapy. Cone beam computed tomography was used to verify both internal and external resorption patterns, whereas clinical and radiographic assessments revealed extensive apical resorption. The patient underwent re-implantation after receiving root canal therapy. Tooth #43 presented a diagnostic challenge, as it appeared non-vital based on sensibility testing. However, a positive response was observed during the test cavity preparation. At the 15-month follow-up, tooth #44 showed clinical and radiographic healing with no visible symptoms of ankylosis or resorption. The sensibility test results for tooth #43 returned to normal, validating the initial false-negative findings.

**Conclusion:**

With a careful technique, appropriate case selection, and thorough follow-up protocols, intentional reimplantation is a legitimate and reliable tooth-preserving treatment option for complex endodontic failures, especially those involving extensive root resorption. This case also emphasizes the value of a thorough diagnostic approach, particularly in cases where the results of the sensibility test conflict with the radiographic and clinical findings in neighboring teeth.

## 1 Introduction

Root resorption, a pathological process that leads to the loss of hard dental tissue, is a significant challenge in endodontic practice. It exists in different forms, broadly categorized as internal or external, each of which has its etiology and presents differently in terms of clinical and radiological manifestations (1). If left untreated, the progressive nature of these lesions often results in extensive tooth structure loss, compromising the long-term prognosis of the tooth and potentially requiring its eventual extraction (1).

Internal root resorption originating within the pulp chamber or root canal is typically asymptomatic in its early phases and often appears as an accidental radiographic finding. Conversely, external root resorption arising from the periodontal ligament (PDL) or surrounding bone can be inflammatory, replacement (ankylosis), or surface-related, with the inflammatory and replacement types posing the most significant threats to tooth longevity (2). Accurate diagnosis and timely intervention are paramount for preventing the progression of these destructive processes (3).

Despite advancements in non-surgical and conventional surgical endodontic techniques, certain complex cases of root resorption remain unresponsive to conventional management. Factors such as the location, extent, and type of resorption, coupled with anatomical constraints such as proximity to vital structures, can severely limit direct access and adequate debridement, thereby hindering successful repair and regeneration (4). In cases where resorption extensively affects the roots, traditional treatment options may be limited, necessitating alternative approaches such as intentional reimplantation (4).

Intentional reimplantation (IR) is a conservative treatment approach for teeth with persistent periapical pathology or complex root morphology for which conventional non-surgical retreatment and apical surgery are not feasible (2). According to Grossman, intentional reimplantation involves the extraction of a tooth, followed by immediate reimplantation, allowing for apical filling of the canals while the tooth is outside the mouth (5). This procedure involves the atraumatic extraction of a tooth, extraoral endodontic management, and reimplantation within a controlled timeframe to preserve the viability of the periodontal ligament (6). It is indicated for teeth with persistent periapical pathology despite previous root canal treatment, particularly when anatomical constraints such as proximity to the maxillary sinus or inferior alveolar nerve limit surgical access. Additionally, it serves as an alternative for managing internal and external root resorption, allowing direct repair of resorptive defects. Other indications include repairable root fractures, inaccessible root perforations, and complex root canal anatomies preventing adequate debridement using conventional methods (7).

The success of IR largely depends on several critical factors, including minimal extra-oral time, atraumatic handling of the PDL, effective decontamination of the root canal system, and proper root end management (8). Although ankylosis and external cervical resorption are potential risks, several studies have demonstrated favorable long-term outcomes, making IR a valuable treatment option for selected cases (8). IR demonstrated favorable survival rates in these studies. Torabinejad reported a weighted mean survival rate of 88% for intentionally replanted teeth in his systematic review (9).

Teeth adjacent to large periapical lesions frequently present a diagnostic dilemma, as inflammatory changes in the surrounding bone and periodontal structures can mimic pulpal involvement despite the pulp remaining vital. Several clinical studies and reviews have demonstrated that periapical radiolucencies often extend beyond the confines of a single tooth apex, sometimes radiographically overlapping neighboring teeth, which may then exhibit altered or even absent responses to sensibility tests (10, 11). However, vitality can be maintained in these adjacent teeth because pulpal necrosis is not an inevitable consequence of periapical inflammation, as the pulp remains protected by intact dentinal and cemental barriers unless there is direct communication or secondary insult (12). This underscores the need for cautious interpretation of sensibility test results and highlights the limitations of relying solely on radiographic evidence when determining pulpal status in teeth located within or near large periapical lesions.

This case report highlights the successful application of IR in managing a complex case of extensive apical root resorption in a mandibular premolar, where conventional endodontic and surgical approaches were not feasible due to anatomical and pathological limitations. It also illustrates the diagnostic challenges posed by an adjacent tooth with overlapping clinical and radiographic findings, emphasizing the importance of a thorough diagnostic evaluation to prevent misdiagnosis and unnecessary treatment. The case reinforces IR as a viable, predictable, and tooth-preserving option for managing refractory endodontic cases, while underscoring the value of comprehensive assessment in ensuring accurate diagnosis, optimal patient care and predictable long-term outcomes.

## 2 Clinical procedures

### 2.1 History and clinical examination

A 25-year-old woman presented to the Post Graduate Clinic seeking completion of a previously initiated root canal treatment on tooth#44. which had been started one week earlier by an undergraduate student. Following the patient's chief complaint of food impaction caused by a fractured tooth.

**Figure 1:**
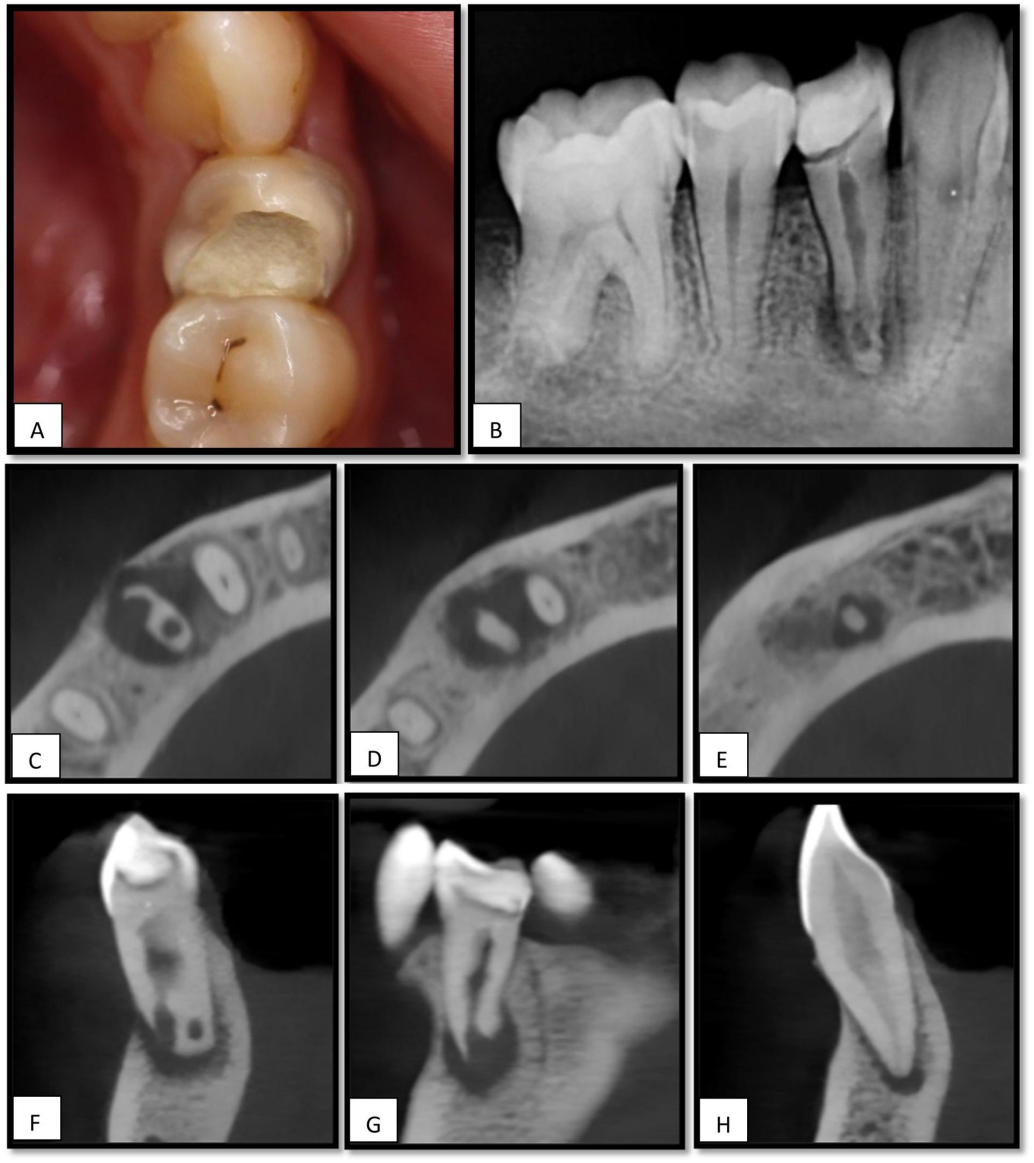
**(A)** Intraoral image for tooth#44. **(B)** intraoral periapical radiograph shows a periapical radiolucency associated with the apex of tooth #44, **(C)** an axial view of CBCT shows apical radiolucency (RL) associated with the apex of tooth #44 with signs of internal and external resorption. **(D,E)**, RL extending to tooth #43. **(F,G)**, sagittal and coronal views for tooth #44 **(H)**, sagittal view for tooth #43 showing apical radiolucency.

The patient was reported to be medically fit, American Society of Anesthesiologists (ASA) class I (13), and had no medications or allergies. The patient's dental history revealed prior orthodontic treatment 6 years previously for 3 years, there was no history of dental trauma. Upon examination, the tooth exhibited no sensitivity to percussion or palpation, no swelling, no sinus tract was observed, the probing depth was normal (2 mm), and both tooth mobility and gingival consistency were normal. Tooth #44 was restored with a temporary filling. Teeth #43 and #45 were present and exhibited normal proximal contact with tooth #44 (Figure 1A). Radiographic assessment revealed periapical radiolucency at the apex of tooth #44 with evidence of root resorption in the apical third of the same tooth (Figure 1B). The crestal bone height was within normal limits.

Tooth 43 was clinically intact and showed no sensitivity to percussion or palpation. Nevertheless, it failed to respond to repeated sensibility testing using both electric pulp testing [Digitest® Digital Pulp Vitality Tester (Parkell Inc., Edgewood, NY, USA)] and cold testing with Endo-Ice F Refrigerant Spray (Coltene, Ref: COH05030, Altstätten, Switzerland) while the contralateral teeth responded normally. Based on these findings, the tooth was considered nonvital.

Tooth #45 exhibited a chalky white appearance at the distal margin and responded with a short, sharp pain on cold testing. The tooth was asymptomatic on percussion or palpation. Radiographic examination revealed a distal coronal radiolucency extending to the middle of the dentin, without any signs of periapical pathology. Based on clinical and radiographic findings, the patient was diagnosed with reversible pulpitis.

Cone-beam computed tomography (CBCT) revealed internal and external root resorption in the apical portion of the root (Figures 1C–G), and radiolucency extending to the apex of tooth # 43 (Figures 1D–H).

The patient presented with two treatment options for tooth #44: (1) extraction followed by dental implant placement, or (2) root canal therapy in conjunction with IR. Surgical endodontic intervention was contraindicated due to anatomical limitations and the extent of apical root resorption, which restricted access and compromised the feasibility of the conventional surgical approach.

Following a comprehensive discussion of the proposed treatment plan, including the nature of the condition, procedural steps, expected outcomes, and potential risks such as postoperative discomfort, failure of reimplantation, ankylosis, and root resorption, the patient decided. The importance of postoperative follow-up and adherence to care instructions was also emphasized. The patient voluntarily provided written informed consent to proceed with root canal therapy followed by IR, and additionally authorized the use of anonymized clinical data and images for academic and publication purposes.

### 2.2 First visit: root canal treatment

**Figure 2:**
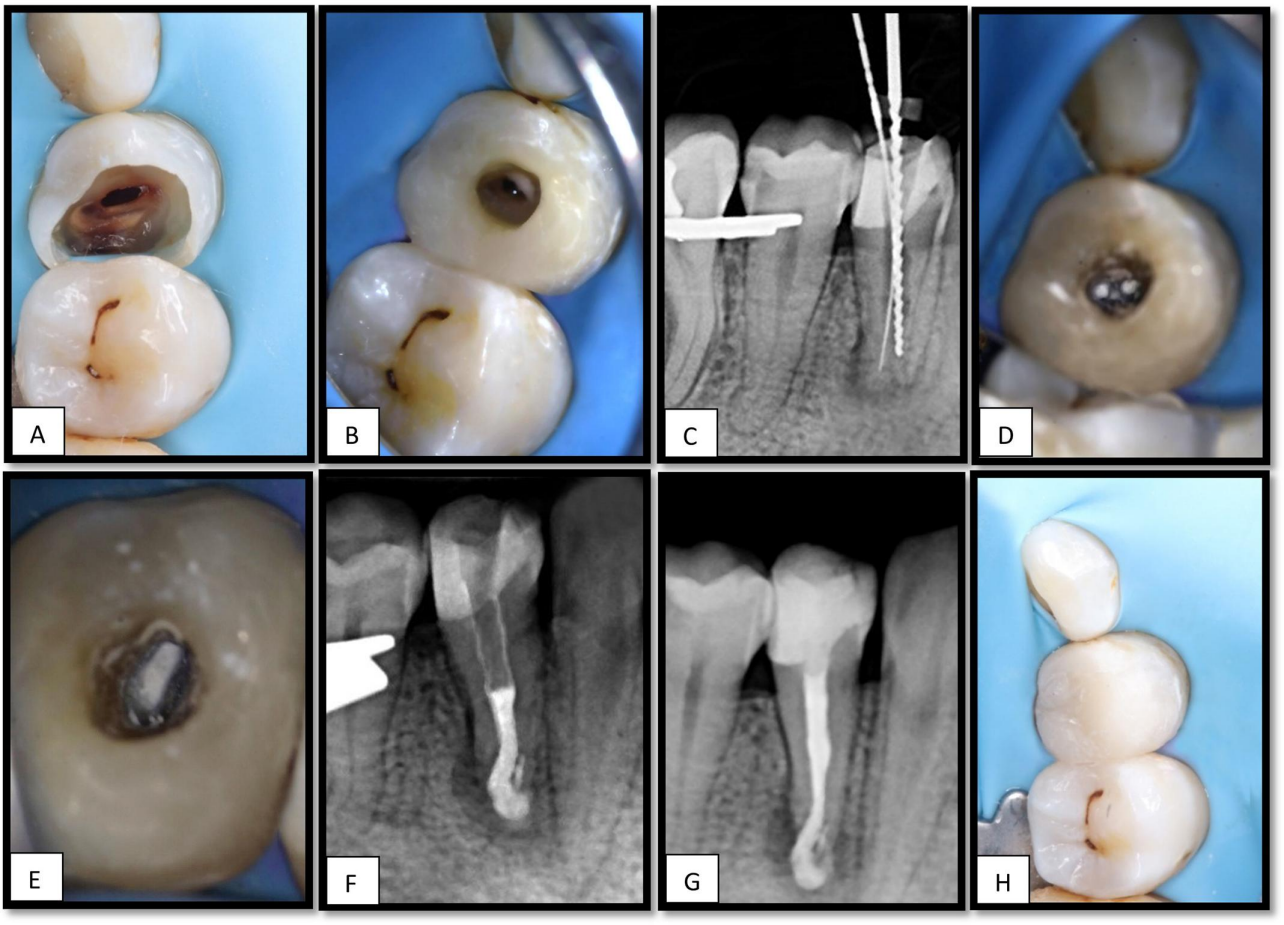
**(A)**, Cavity after caries excavation. **(B)**, Access cavity refinement after coronal build-up. **(C)**, Working length determination using size 10 K-file in the lingual canal and size 50 H-file in the buccal canal. **(D)**, MTA apical plug in both canals, **(E)**, Thermoplastic Obturation of the remaining canal space done till the CEJ, **(F)**, PA showing the apical plug with slight extrusion of the material. **(G)**, PA showing the obturation of #44, **(H)**, Composite resin restoration placed.

The patient underwent an inferior alveolar nerve block using 2% lidocaine with 1:100,000 epinephrine. Rubber dam isolation was performed, and the temporary restoration was removed. Caries excavation was completed (Figure 2A), after which a Teflon tape was placed over the canal orifice to protect it during coronal build-up with the composite resin. Subsequently, the access cavity was refined (Figure 2B) under magnification using a CJ-Optik Flexion (CJ-Optik, Wetzlar, Germany). Examination revealed a single orifice bifurcating into two distinct canals in the middle third of the root.

The working length was determined using an electronic apex locator (EAL) in combination with an intraoral periapical radiograph. A 10 K-file was positioned 17 mm from the lingual canal, whereas a 50 H-file reached 15.5 mm from the buccal canal (Figure 2C).

Canal preparation was performed using the ProTaper Ultimate system, sequentially employing the shaper and F1 and F2 files. Minimal manual instrumentation with 40 H-files was performed for the buccal canal. The final irrigation included 2% sodium hypochlorite (NaOCl) with activation using an ultrasonic device (Irri Safe tip) for three cycles, followed by 5 mL of 17% EDTA for 1 min, and a final rinse with 2% NaOCl.

Calcium hydroxide [Ca(OH)₂] was not employed in this case. The decision was based on the extent of apical root resorption, which required immediate apical sealing with mineral trioxide aggregate (MTA) and subsequent intentional reimplantation, rather than attempting to arrest resorption with intracanal medicaments. Obturation was achieved by placing a mineral trioxide aggregate with conventional setting (ProRoot MTA, Dentsply Sirona, USA) in the apex 6 mm of both canals (Figures 2D,E). A slight accidental extrusion of the material occurred (Figure 2F). The remaining canal spaces were backfilled with thermoplasticized gutta-percha (Figure 2G), and the final composite resin restoration was placed (Figure 2H) and the patient was scheduled for surgical intervention.

### 2.3 Second visit: surgical day

**Figure 3:**
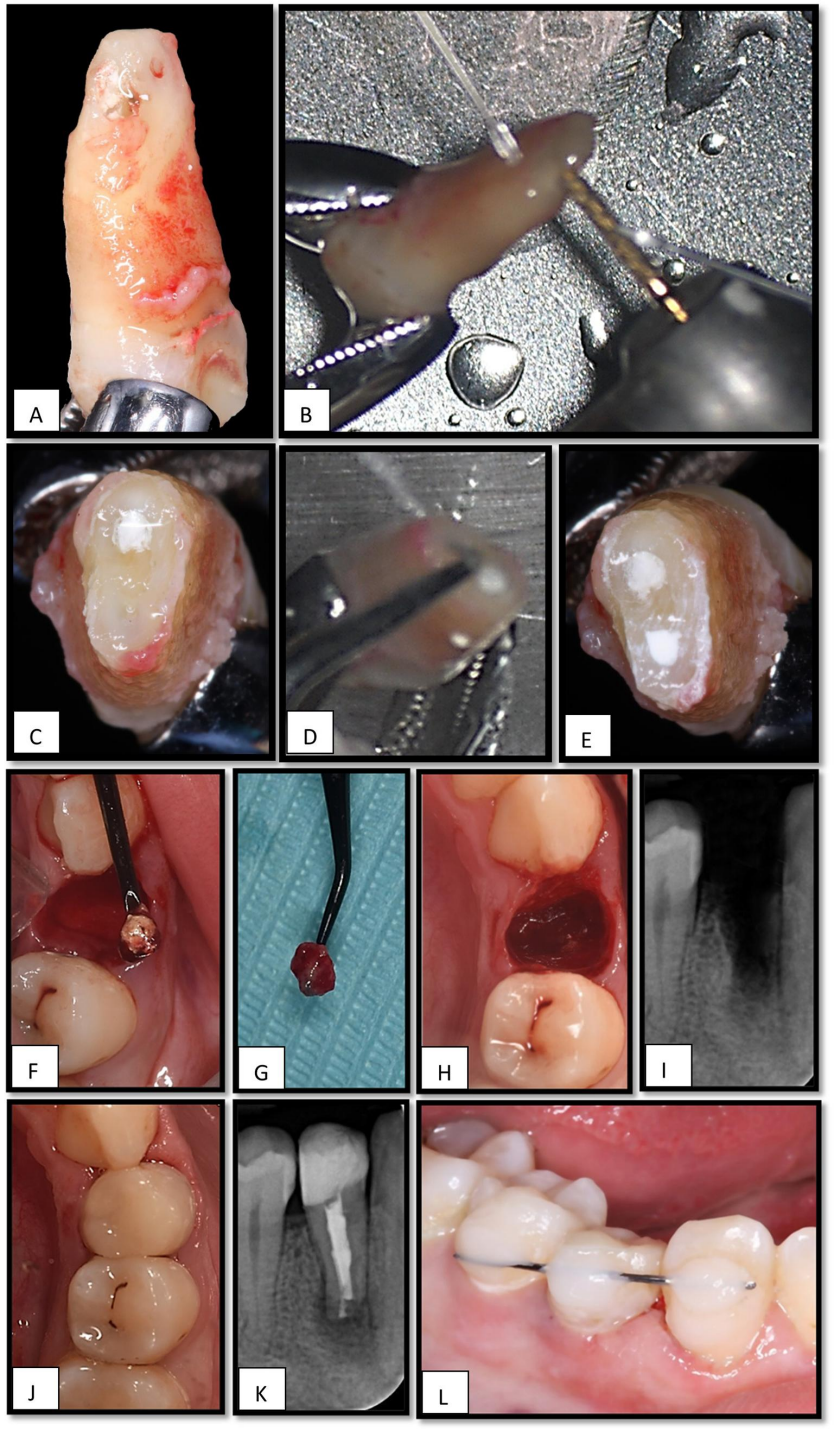
**(A)**, Tooth # 44 extracted using lower premolar forceps, **(B)**, cutting the apical 4 mm by high-speed straight fissure diamond bur, **(C)**, the lingual canal has insufficient MTA filling, **(D)**, preparing the apical 3 mm from the lingual canal by ultrasonic, **(E)**, retrograde filling using MTA pro-root in the lingual canal. **(F)**, Extruded root filling material is curetted. **(G)**, Granulation tissue is removed from the socket. **(H)**, Intraoral view of the extraction socket before reinsertion. **(I)**, Periapical radiograph of an empty socket related to tooth #44. **(J)**, Post-operative clinical view after reimplantation of tooth #44, **(K)**, Periapical x-ray shows the tooth #44 after reimplantation, **(L)**, Tooth is stabilized using flexible splint (orthodontic wire) and composite resin.

The patient reported remaining asymptomatic after completion of the single-visit endodontic treatment. At the second visit, 5 days after the RCT, the patient was instructed to rinse with 0.12% chlorhexidine gluconate and received 400 mg of ibuprofen as premedication. Two surgeons performed the procedure. Following standard surgical preparation, profound anesthesia was achieved via an inferior alveolar and lingual nerve block and buccal infiltration using 2% lidocaine with 1:100,000 epinephrine. The mucoperiosteal flap was carefully reflected to expose the apical portion of the crown margin. The tooth was then gently extracted using forceps with contact placed above the cemento-enamel junction (CEJ) to ensure minimal trauma to the surrounding tissues (Figure 3A).

Immediately after extraction, the extraoral phase was initiated and timed. The extracted tooth was handled gently with taped forceps and rinsed with sterile saline to remove debris without desiccating or scraping the root surface under magnification using a CJ-Optik Flexion (CJ-Optik, Wetzlar, Germany). The apical third was examined, and a 4 mm apical resection was performed using a high-speed handpiece under copious irrigation to eliminate the area of resorption (Figures 3B,C).

A retrograde cavity, approximately 3 mm in depth, was prepared in the lingual canal using a diamond-coated ultrasonic tip (ED10D; Satelec Acteon, Mérignac, France) (Figure 3D) and filled with TotalFill RRM Fast-Set Putty (FKG Dentaire, Switzerland)to ensure a three-dimensional apical seal (Figure 3E).

While the tooth was undergoing extraoral treatment, the second surgeon gently curetted the apical portion of the extraction socket to remove the extruded material (Figure 3F) and granulation tissue (Figure 3G), taking care not to disturb the socket walls coronal to the apex (Figures 3H,I).

Care was taken to limit the total extra-oral time to less than 10 min. Once the root-end procedure was completed, the tooth was carefully reinserted into the original socket (Figure 3J) to ensure the correct orientation and passive fit. Subsequently, a flexible splint using an orthodontic wire and composite resin was placed to stabilize the tooth and permit physiological mobility during healing (Figures 3K,L).

The patient was prescribed analgesics and instructed to continue rinsing twice daily with 0.12% chlorhexidine gluconate for 1 week. The splint was removed after 2 weeks. Clinical and radiographic follow-ups were scheduled for 1, 3, and 6 months, and 1 year to monitor healing and assess signs of ankylosis or root resorption.

For tooth#43, a cavity test was performed under rubber dam isolation and without local anesthesia, to accurately assess pulpal responsiveness. The drilling started in enamel and extended into approximately 1–1.5 mm inside the dentin then the patient experienced a sudden and sharp pain response, indicating persisting neural activity within the pulp. Thus, the endodontic treatment was deferred, and the cavity was filled with composite resin restoration, and the tooth was kept under observation. Tooth #45, which was diagnosed with reversible pulpitis, was managed conservatively and restored with a Class II composite restoration.

Clinical and radiographic follow-ups, including periapical radiographs and CBCT scans over 15 months, demonstrated successful periapical healing, functional stability, and the absence of progressive resorptive changes. This case highlights the effectiveness of IR as a tooth-preserving strategy for complex resorptive cases.

**Figure 4:**
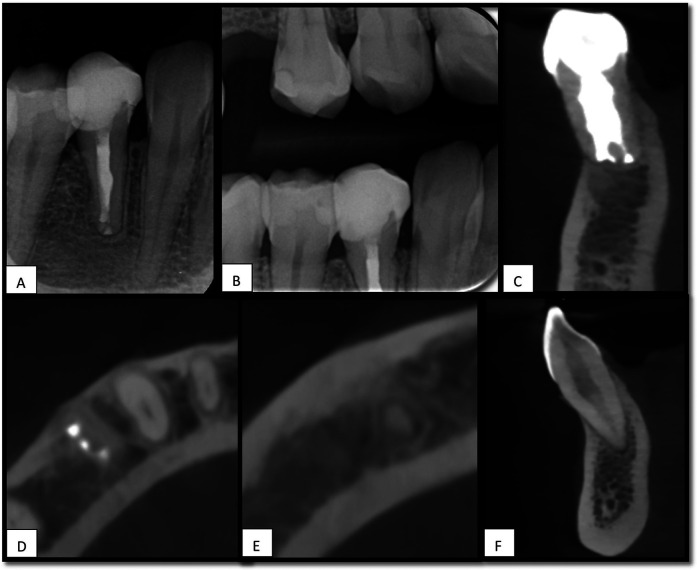
15 months follow-up, **(A)**, periapical x-ray for tooth #44, shows healing of apical radiolucency. **(B)**, Bitewing x-ray. **(C)**, Sagittal view of CBCT for tooth #44 shows no signs of root resorption. **(D,E)**, Axial view of CBCT shows healing of periapical area associated with teeth #44 and #43. **(F)**, Sagittal view shows no RL associated with tooth #43.

Follow-up evaluations demonstrated recovery of normal pulpal sensibility in tooth #43, a mild response to cold testing, and a positive response to electric pulp test, accompanied by complete periapical bone regeneration. CBCT imaging revealed signs of healing in the previously detected periapical radiolucency associated with tooth #43, which was observed before treatment for tooth #44 (Figure 4).

## 3 Discussion

This case report presents a 15-month follow-up of IR for the management of a mandibular premolar (44) exhibiting extensive apical root resorption. The clinical and radiographic outcomes demonstrated favorable healing without evidence of ankylosis or root resorption, supported by normal tooth mobility, physiological response to percussion, and radiographic demonstration of an intact periodontal ligament space. Although many resorption-related complications tend to manifest within the first year post-replantation (14), long-term monitoring remains essential to capture late-onset complications (15).

Intentional reimplantation was performed immediately after root canal therapy due to critical anatomical and procedural factors. The lingual canal was inaccessible, limiting debridement and resulting in insufficient obturation, compounded by extrusion of material and radiographic evidence of extensive apical resorption. Although calcium hydroxide [Ca(OH)₂] is often used to improve disinfection in such cases, it was not applied here because immediate apical sealing with MTA and reimplantation offered the most predictable approach for achieving a three-dimensional apical seal and preventing persistent infection.

In the present case, surgical endodontic treatment was contraindicated because of the restricted surgical access and the extent of apical resorption, which led to the selection of IR as a conservative alternative to extraction and implant placement. The literature supports high survival rates after IR, with the weighted mean survival rate of intentionally replanted teeth reaching 88%.In the current case, an effort was made to limit the extraoral phase to under 10 min, well below the reported 15-minute threshold that is considered critical for achieving favorable healing outcomes without complications (9).

The use of a two-operator protocol, magnification with a dental microscope, continuous hydration of the root surface, and atraumatic manipulation are critical for minimizing damage to the periodontal ligament. Careful socket debridement was performed concurrently to remove the extruded filling material and granulation tissue while preserving the socket architecture. Root-end resection of 4 mm was performed to eliminate the apical resorptive defect, followed by ultrasonic retrograde preparation and filling of the lingual canal with ProRoot MTA, ensuring a hermetic seal and sufficient filling of the buccal canal with MTA during orthograde filling. The atraumatic extraction technique was achieved with conventional forceps and its placed above the CEJ because improper placement of forceps at or below the CEJ may damage the periodontal ligament or cervical root surface, increasing the risk of external cervical resorption and compromising long-term prognosis (16). Previous studies have indicated that minimizing mechanical compression on the root surface during extraction reduces the risk of cementoblast damage, which is strongly associated with replacement resorption and ankylosis (16).

In the present case, the diagnostic complexity of tooth #43 highlights the challenges of assessing teeth adjacent to necrotic teeth with extensive periapical pathology. Although sensibility testing initially yielded a negative response, subsequent diagnostic cavity preparation confirmed pulp vitality, and follow-up demonstrated recovery of a normal response. This finding is consistent with reports that adjacent teeth encompassed radiographically by large periapical lesions can maintain vital pulps despite localized inflammatory changes in periradicular tissues (10–12). Such false-negative responses are most likely attributable to transient neurovascular impairment or periapical inflammatory pressure rather than true pulpal necrosis (11, 17).

The sudden pain response during dentin preparation of tooth #43 indicated persisting neural activity, suggesting that the pulp was not completely necrotic, although partial necrosis cannot be excluded. Since sensibility tests (cold and EPT) evaluate neural rather than vascular status, they are prone to misclassification, particularly in teeth adjacent to inflamed periapical regions. Several mechanisms can contribute to false-negative outcomes: hypoxia and pressure affecting A-delta fibers at the apical neurovascular bundle while C-fibers remain functional; transient impairment of A-delta conduction by inflammatory mediators and periapical edema; and attenuation of stimulus transfer due to dentine sclerosis or altered fluid dynamics (18). Reported diagnostic performance indicates that the cold test has pooled sensitivity of ∼0.87 and specificity of ∼0.84, whereas EPT has lower sensitivity (∼0.72) but higher specificity (∼0.93), making it more reliable for ruling in vitality (19). By contrast, vitality tests that assess pulpal blood flow, such as laser Doppler flowmetry (LDF) and pulse oximetry (PO), show superior accuracy, with pooled sensitivity and specificity values of ∼0.98/0.95 for LDF and ∼0.97/0.95 for PO, while recent reviews of PO report mean sensitivity/specificity around 95%/99% (18). These methods reduce subjectivity and are less affected by neural dysfunction, though their limited availability and technique sensitivity hinder routine use. Therefore, the initial non-responsiveness of tooth #43 to cold and EPT most likely reflected a transient false-negative caused by periapical inflammation impairing A-delta fiber function, with recovery evident once the local neurovascular environment stabilized. Conservative monitoring combined with a multimodal diagnostic approach is essential to avoid unnecessary root canal treatment of vital teeth, provided that patients are followed with regular re-evaluation (17, 20).

The sudden pain response during access preparation provided additional support for the pressure hypothesis. Larger myelinated A-type nerve fibers are more sensitive to pressure and hypoxia than unmyelinated C-type fibers (18). Although the pulpal response to electrical and cold sensibility testing, typically mediated by A-fibers, may have diminished, the combination of heat build-up and mechanical stimulation from the cutting bur likely triggered a delayed pain response via C-fiber activation (21).

At the 15-month follow-up, the neighboring tooth#43, which initially presented with apical radiolucency and no response to cold or EPT tests, demonstrated periapical healing and regained responsiveness to sensibility testing, in the absence of root resorption or coronal discoloration. These findings provide supportive—though not definitive—evidence of pulp health. Because the sensibility tests assess neural response rather than true pulp vitality, and that confirmation of vascular status would require additional methods such as laser Doppler flowmetry, pulse oximetry, or histologic analysis, which were not performed in this case (22). The initial irresponsiveness may be attributed to the activation of bone resorption processes and physiological changes in the adjacent tissues (23).These findings suggest that the lesion associated with tooth #43 likely represented resolving apical periodontitis secondary to localized inflammation around the tooth root, causing bone resorption, and the body requires time to repair the damage (24).

## 4 Conclusion

Within the limitations of this report, this case supports the role of IR as a valid tooth-preserving option in complex resorptive cases, when meticulous techniques, proper case selection, and comprehensive follow-up protocols are employed.

Furthermore, it can be difficult and misleading to diagnose the pulp vitality of unaffected teeth, whose apices extend into the nearby lesion, and alterations in the nerve bundle may prevent vital teeth from responding to neural-based sensibility tests, producing false-negative results. Therefore, laser Doppler flowmetry and pulse oximetry tests are recommended to guarantee a more accurate initial diagnosis under such circumstances.

## Data Availability

The original contributions presented in the study are included in the article/Supplementary Material, further inquiries can be directed to the corresponding author.
